# Analysis of a Machine Learning–Based Risk Stratification Scheme for Chronic Limb-Threatening Ischemia

**DOI:** 10.1001/jamanetworkopen.2022.3424

**Published:** 2022-03-22

**Authors:** Jayer Chung, Nikki L. B. Freeman, Michael R. Kosorok, William A. Marston, Michael S. Conte, Katharine L. McGinigle

**Affiliations:** 1 Division of Vascular Surgery and Endovascular Therapy, Michael E. DeBakey Department of Surgery, Baylor College of Medicine, Houston, Texas; 2 Department of Biostatistics, University of North Carolina at Chapel Hill, Chapel Hill; 3 Division of Vascular Surgery, Department of Surgery, University of North Carolina at Chapel Hill, Chapel Hill; 4 Department of Surgery, University of California at San Francisco, San Francisco

## Abstract

**Importance:**

Valid risk stratification schemes are key to performing comparative effectiveness research; however, for chronic limb-threatening ischemia (CLTI), risk stratification schemes have limited efficacy. Improved, accurate, comprehensive, and reproducible risk stratification models for CLTI are needed.

**Objective:**

To evaluate the use of topic model cluster analysis to generate an accurate risk prediction model for CLTI.

**Design, Setting, and Participants:**

This multicenter, nested cohort study of existing Project of Ex Vivo Vein Graft Engineering via Transfection (PREVENT) III clinical trial data assessed data from patients undergoing infrainguinal vein bypass for the treatment of ischemic rest pain or ischemic tissue loss. Original data were collected from January 1, 2001, to December 31, 2003, and were analyzed in September 2021. All patients had 1 year of follow-up.

**Exposures:**

Supervised topic model cluster analysis was applied to nested cohort data from the PREVENT III randomized clinical trial. Given a fixed number of clusters, the data were used to examine the probability that a patient belonged to each of the clusters and the distribution of the features within each cluster.

**Main Outcomes and Measures:**

The primary outcome was 1-year CLTI-free survival, a composite of survival with remission of ischemic rest pain, wound healing, and freedom from major lower-extremity amputation without recurrent CLTI.

**Results:**

Of the original 1404 patients, 166 were excluded because of a lack of sufficient feature and/or outcome data, leaving 1238 patients for analysis (mean [SD] age, 68.4 [11.2] years; 800 [64.6%] male; 894 [72.2%] White). The Society for Vascular Surgery Wound, Ischemia, and Foot Infection grade 2 wounds were present in 543 patients (43.8%), with rest pain present in 645 (52.1%). Three distinct clusters were identified within the cohort (130 patients in stage 1, 578 in stage 2, and 530 in stage 3), with 1-year CLTI-free survival rates of 82.3% (107 of 130 patients) for stage 1, 61.1% (353 of 578 patients) for stage 2, and 53.4% (283 of 530 patients) for stage 3. Stratified by stage, 1-year mortality was 10.0% (13 of 130 observed deaths in stage 1) for stage 1, 13.5% (78 of 578 patients) for stage 2, and 20.2% (105 of 521 patients) for stage 3. Similarly, stratifying by stage revealed major limb amputation rates of 4.2% (5 of 119 observed major limb amputations in stage 1) for stage 1, 10.8% (55 of 509 patients) for stage 2, and 18.4% (81 of 440 patients) for stage 3. Among survivors without a major amputation, the rates of CLTI recurrence were 9.2% (11 of 119 observed recurrences in stage 1) for stage 1, 24.9% (130 of 523 patients) for stage 2, and 29.6% (132 of 446 patients) for stage 3.

**Conclusions and Relevance:**

The topic model cluster analysis in this cohort study identified 3 distinct stages within CLTI. Findings suggest that CLTI-free survival is an end point that can be accurately and reproducibly quantified and may be used as a patient-centric outcome.

## Key Points

**Question:**

At the time of chronic limb-threatening ischemia (CLTI) diagnosis, how are increasing patient risk, wound severity, and degree of atherosclerotic occlusions associated with CLTI-free survival?

**Findings:**

This cohort study analyzed nested cohort data for 1238 patients from the Project of Ex Vivo Vein Graft Engineering via Transfection (PREVENT) III trial, the largest prospective randomized clinical trial evaluating the efficacy of edifoligide on outcomes in infrainguinal bypass for CLTI. The study identified 3 distinct clusters within the cohort, with 1-year CLTI-free survival rates of 82.3% for stage 1, 61.1% for stage 2, and 53.4% for stage 3.

**Meaning:**

These findings suggest that, based on presenting characteristics, there are distinct clusters of patients with different 1-year CLTI-free survival; therefore, CLTI-free survival can be accurately and reproducibly quantified and may be used as a patient-centric outcome.

## Teaser

This cohort study uses topic model cluster analysis to evaluate a risk stratification scheme for chronic limb-threatening ischemia.

## Introduction

Peripheral arterial disease is a common and debilitating manifestation of cardiovascular disease that impairs blood flow to the lower extremities. Chronic limb-threatening ischemia (CLTI) is the most severe manifestation of peripheral arterial disease and encompasses all patients with objective evidence of arterial insufficiency and symptoms of ischemic pain at rest and/or tissue loss (ulcerations, wounds, or gangrene). Required intensive medical therapy, revascularization, and/or wound management for patients with CLTI is complex, with highly variable patient responses and frequent reinterventions and even hospitalizations per year. Outcomes for patients with CLTI remain suboptimal despite evolving devices, medical therapeutics, and modern surgical techniques. Comparative effectiveness research (CER) is essential to iteratively improve data-driven outcomes in CLTI, yet the overwhelming heterogeneity of treatments and patient presentations in CLTI stymies CER by preventing objective outcome comparisons.^1,2^ Patients and physicians have limited high-quality data to balance the competing mortality risk, limb threat, procedural invasiveness, hemodynamic efficacy, and longevity of a proposed procedure for a given patient with CLTI.^1,2^

Although multiple researchers have developed baseline risk stratification schemes to enable CER in CLTI, none have been universally adopted.^2^ All have modest predictive ability in CLTI, with model accuracy less than 72%.^1,2^ No prior risk stratification model has been prospectively validated against large external data sets. End points of prior models are variable, often focusing on limb outcomes or survival. Few risk stratification schemes use modern computational advances, such as cluster analyses and/or machine learning to optimize model accuracy.^3,4^ The Society for Vascular Surgery Wound, Ischemia, and Foot Infection (WIfI) score has been validated with regard to limb-based outcomes in retrospective cohorts.^5,6^ Unfortunately, WIfI was designed to predict major amputation at only 1 year and is not understood with regard to mortality or other non–limb-specific composite outcomes.

Moreover, efficacy of infrainguinal bypass has been challenged over the past 2 decades because of the penetrance of endovascular devices and techniques.^7^ Indeed, training patterns suggest that experience with infrainguinal bypass and in particular infrageniculate bypasses, may be waning.^8^ No one is certain whether current outcomes reflect ideal infrainguinal bypass outcomes, and the comparisons between endovascular and infrainguinal bypass outcomes are potentially biased by this unmeasured confounding. Hence, it remains germane to model CLTI outcomes after infrainguinal bypass within a CLTI cohort in which the outcomes reflect what is truly achievable with infrainguinal bypass. Finally, only a small amount of literature focuses on patient-centric outcomes in CLTI. For example, outcomes analyses in CLTI focus frequently on amputation-free survival or patency. Although important, these outcomes fail to capture patient-centric outcomes, such as relief of ischemic rest pain and wound healing.

In this context, we aim to recapitulate our novel technique to generate a machine learning–based risk stratification scheme for CLTI within the nested cohort data from the previously published Project of Ex Vivo Vein Graft Engineering via Transfection (PREVENT) III trial.^4,9^ We also aim to evaluate the ability to accurately and reproducibly use CLTI-free survival as an end point in CLTI outcomes research.

## Methods

### Study Population

The PREVENT III randomized clinical trial comprised 1404 patients undergoing infrainguinal vein bypass for the treatment of ischemic rest pain or ischemic tissue loss.^9^ Patients participating in the trial, which was monitored by a data and safety monitoring board, provided written informed consent on enrolling in the trial. In our cohort, we included all patients who had complete trial enrollment and outcome data. Original data were collected from January 1, 2001, to December 31, 2003, and were analyzed in September 2021. All patients had 1 year of follow-up. Nested cohort data from the PREVENT III trial were anonymized before data analysis. Because of the retrospective nature of the analysis and impracticality of obtaining patient consent years after the closure of the clinical trial, the institutional review board at the University of North Carolina waived consent. The study followed the Strengthening the Reporting of Observational Studies in Epidemiology (STROBE) reporting guideline.^10^

### Outcomes

The primary outcome measured was 1-year CLTI-free survival, which is a composite end point that includes patients who are alive with remission of CLTI symptoms (wound healing and/or freedom from ischemic pain), no ipsilateral major limb amputation, and no recurrence of ipsilateral CLTI at 1 year. Secondary outcomes are the individual components of this composite end point. We considered 1-year CLTI-free survival as a binary outcome, and we classified patients as having 1-year CLTI-free survival if they were alive, were free of major lower-extremity amputation, were free of recurrence, and had healed their index wound at 1 year after the index procedure. Specifically, mortality, major limb amputation, and recurrence of CLTI and their respective dates were recorded in the case report forms and used directly from the trial data set. For patients who had an index wound, we used the follow-up wound examination records to determine whether the index wound was healed. If the index wound was recorded as healed before or at 1 year after the index procedure, the wound was considered healed. If it was recorded as unhealed at 1 year after the index procedure, the wound was considered unhealed.

For the wound grade of the WIfI score, we used data from the baseline examination of the index leg. Records indicated the presence of gangrene and/or ulcers for each toe, the forefoot, the hindfoot, or other location. Free text responses to wound locations coded as other were recoded to the appropriate corresponding category (toe[s], forefoot, hindfoot, or leg). Wound scores were assigned as follows: wound grade 0, no index wound; wound grade 1, an ulcer was reported on only 1 toe; wound grade 2, an ulcer was reported on multiple toes OR gangrene was reported for 1 or more toes or the forefoot; wound grade 3, an ulcer was reported on the hindfoot or leg OR gangrene was reported for the forefoot, hindfoot, or leg; and highest score, multiple wounds (eg, a toe ulcer [grade 1] and a gangrenous hindfoot [grade 3]).

### Statistical Analysis

Demographic, comorbid, and CLTI-related characteristics for the cohort studied were described using medians (IQRs) for continuous variables and numbers (percentages) for categorical and binary variables. In accordance with the STROBE statement for reporting outcomes from observational studies, we do not include *P* value comparisons between the 2 groups in our description.^10^

Using the strategy developed in McGinigle et al,^4^ we used topic modeling from the natural language processing literature to cluster patients. Topic models are used to model the words in a corpus of documents to discover latent topics within those documents. In many cases, documents may have multiple topics; for example, movie reviews may be clustered by movie genre (eg, drama or horror) or by whether it was a positive review or not. Supervision is introduced to the model to guide topic formation so that topics are predictive of the desired response (eg, movie genre). In the context of our work, we considered our sample as a corpus of documents, with each individual in the sample as a document and the comorbid and CLTI-related features of each individual as the words in a document; supervision is provided by the outcome of interest, which in our case is CLTI-free survival.

To learn the latent topics or clusters in our sample, we specified a supervised latent Dirichlet allocation model,^11^ which models responses, latent topics, documents, and words within documents. The generative model supposes that documents are generated from a set of latent topics and those topics induce an unknown distribution over the vocabulary. Thus, each topic has words that are more likely or less likely to appear in a document that arose from that topic. Translating from text documents to patients with CLTI, this means that patients in a particular cluster are more likely to share common features (common vocabulary), for example, wound grade and history of myocardial infarction, than patients in a different cluster. In addition to looking for common patterns, the latent clusters are formed with supervision from the patient outcome of 1-year CLTI-free survival. Specifically, CLTI-free survival enters the model as conditional on the proportion of features that are assigned to each cluster for the patient; we use a probit model, a type of generalized linear model for regression with binary outcomes. Given a fixed number of topics or clusters, we use the data to learn the probability that a patient belongs to each of the clusters and the distribution over the features within each cluster.

We slightly modified the model so that the regression for the response included demographic variables, such as sex and race, that were not included in the features used for topic formation, because demographic characteristics are likely to be associated with differential response owing to systemic disparities in health and health care but are not features on which we would want to use to characterize CLTI subtypes. To learn the model parameters, we implemented the collapsed Gibbs sampling approach described by Griffiths and Steyvers^12^ and used the posterior samples of the topic assignments to calculate sufficient statistics of the model parameters.

The data were analyzed using *K = 2,…,9* clusters. The number of clusters in the final model was 3 because there was a clear point of diminishing returns for adding more clusters, and clinician examination of the patient features associated with each cluster confirmed a practical clinical applicability. In addition, we used bayesian model selection to approximate the posterior probability of the observed data (features) given that the model in the study by Griffiths and Steyvers^12^ and the model with *K* = 3 clusters yielded the highest posterior probability. After selecting the model with 3 clusters, the probabilities of patients belonging to a particular cluster were also computed. We considered patients to be in stage 1 if the probability of them being in stage 1 was higher than the probability of them being in stage 2 or stage 3 and similarly for stages 2 and 3. Clusters were characterized by the features with which they were most often associated and further characterized by analyzing the characteristics of the patients within them.

For this hypothesis-generating study, we emphasize that our model describes our cohort and provides insight into the feasibility of delineating limb-based and survival-based stages of CLTI. Based on guidance from the American Statistical Association, we did not conduct null hypothesis significance tests to compare the latent stages discovered as one may in a formal confirmatory study.^13,14,15^ Analyses were performed using R, version 4.0.4 (R Foundation for Statistical Computing) and Julia, version 1.6 (MIT Lincoln Laboratory).

## Results

Of the 1404 patients originally randomized in PREVENT III, 166 patients were excluded because of a lack of significant feature and/or outcome data, leaving 1238 patients (mean [SD] age, 68.4 [11.3] years; 800 [64.6%] male; 10 [0.8%] Asian, 221 [17.9%] Black, 96 [7.8%] Hispanic, 894 [72.2%] White, and 17 [1.4%] other [no further detail available from the trial data set]) for analysis. The most prevalent comorbidities were hypercholesterolemia (680 patients [54.9%]) and diabetes (800 patients [64.6%]). With respect to presentation severity, 645 patients (52.1%) presented with rest pain, and 313 (25.3%) presented with a combination of rest pain and ischemic wounds. A total of 542 patients (43.8%) presented with a grade 2 wound as per the WIfI classification.^5^ Most bypasses (680 patients [54.9%]) were performed to a tibial artery target (Table 1).

**Table 1.** Characteristics of the Patient Cohort

Characteristic	Finding^a^
Age, mean (SD) [range], y	68.4 (11.3) [30-99]
Sex	
Female	438 (35.4)
Male	800 (64.6)
Race	
Asian	10 (0.8)
Black	221 (17.9)
Hispanic	96 (7.8)
White	894 (72.2)
Other^b^	17 (1.4)
Baseline weight, kg	78.7 (17.7)
Rest pain	645 (52.1)
Wound	
Grade 1	287 (23.2)
Grade 2	543 (43.8)
Grade 3	313 (25.3)
Stroke	245 (19.8)
Previous cardiac procedure	426 (34.4)
Diet- or medication-controlled diabetes	384 (31.0)
Insulin-controlled diabetes	416 (33.6)
Hypercholesterolemia or hyperlipidemia	680 (54.9)
Smoker	
Former	605 (48.9)
Current	321 (25.9)
Never	312 (25.2)
Popliteal bypass target	
Above knee	129 (10.4)
Below knee	279 (22.5)
Bypass target	
Tibial	680 (54.9)
Pedal	150 (12.1)
Previous procedure	
In the index leg	328 (26.5)
In the nonindex leg	316 (25.5)

^a^ Data are presented as number (percentage) of patients unless otherwise indicated.

^b^ No other information provided in the data set.

### Feature Differences in Each Stage Identified by Cluster Analysis of CLTI-Free Survival

**Figure 1.**
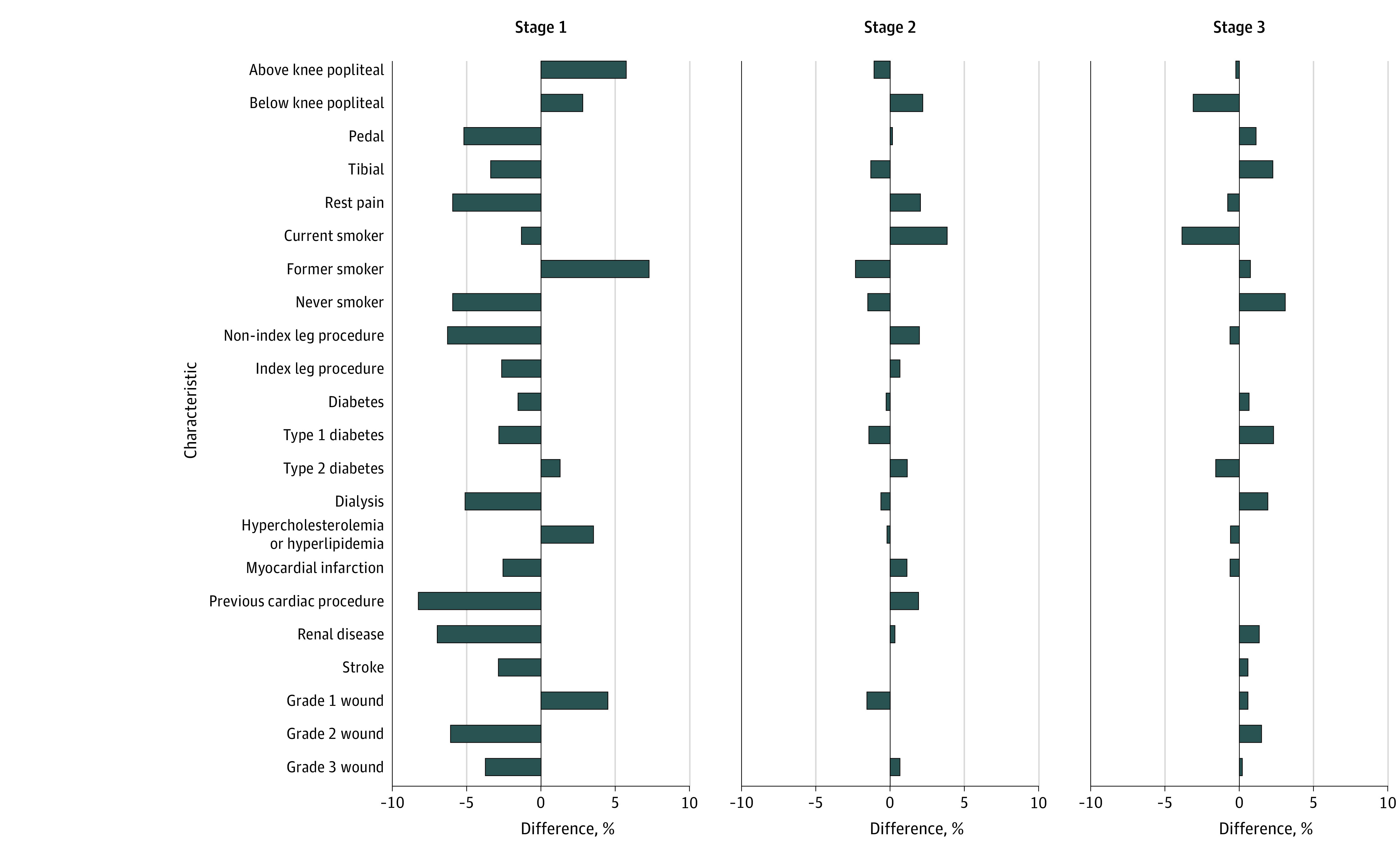
Characteristics of Each Stage as a Percentage Difference From the Mean Cohort Difference was calculated as stage minus study sample.

Overall CLTI-free survival at 1 year was 60.0% (743 patients). We identified 3 clusters within the nested cohort (130 patients clustered in stage 1, 578 in stage 2, and 530 in stage 3). Results of our cluster analysis revealed 1-year CLTI-free survival as follows: 82.3% (107 of 130 patients) at stage 1, 61.1% (353 of 578 patients) at stage 2, and 53.4% (283 of 530 patients) at stage 3. Patients were considered to be in stage 1 if the probability of them being in stage 1 was higher than the probability of being stage 2 or stage 3 and similarly for stage 2 and stage 3. Of the 130 patients clustered into stage 1, 113 (86.9%) had an index wound. Of the 578 patients clustered into stage 2, 528 (91.4%) had an index wound. Among the 530 patients in stage 3, 501 (94.5%) had an index wound. Other significant differences between each stage are numerically quantified in Table 2 and graphically represented in Figure 1.

**Table 2.** Prevalence of Each Feature Within Each Stage

Feature	No. (%) [95% CI]		
	Stage 1	Stage 2	Stage 3
Wound			
Grade 3	28 (21.5) [14.5-28.6]	157 (26.0) [22.4-29.5]	135 (25.5) [21.8-29.2]
Grade 2	49 (37.7) [29.4-46.0]	253 (43.8) [39.7-47.8]	240 (45.3) [41.1-49.5]
Grade 1	36 (27.7) [20.0-35.4]	125 (21.6) [18.3-25.0]	126 (23.8) [20.2-27.4]
Stroke	22 (16.9) [10.5-23.4]	115 (19.9) [16.6-23.2]	108 (20.4) [17.0-23.8]
Renal disease	24 (18.5) [11.8-25.1]	149 (25.8) [22.2-29.3]	142 (26.8) [23.0-30.6]
Previous cardiac procedure	34 (26.2) [18.6-33.7]	210 (36.3) [32.4-40.2]	182 (34.3) [30.3-38.4]
Myocardial infarction	35 (26.9) [19.3-34.6]	177 (30.6) [26.9-34.4]	153 (28.9) [25.0-32.7]
Hypercholesterolemia or hyperlipidemia	76 (58.5) [50.0-66.9]	316 (54.7) [50.6-58.7]	288 (54.3) [50.1-58.6]
Dialysis	9 (6.9) [2.6-11.3]	66 (11.4) [8.8-14.0]	74 (14.0) [11.0-16.9]
Type 2 diabetes	42 (32.3) [24.3-40.4]	186 (32.2) [28.4-36.0]	156 (29.4) [25.6-33.3]
Type 1 diabetes	40 (30.8) [22.8-38.7]	186 (32.2) [28.4-36.0]	190 (35.8) [31.8-39.9]
Diabetes	9 (63.01) [54.8-71.4]	66 (64.4) [60.5-68.3]	74 (65.3) [61.2-69.3]
Procedure			
Index leg	31 (23.8) [16.5-31.2]	157 (27.2) [23.5-30.8]	140 (26.4) [22.7-30.2]
Nonindex leg	25 (19.2) [12.5-26.0]	159 (27.5) [23.9-31.2]	132 (24.9) [21.2-28.6]
Smoker			
Never	25 (19.2) [12.5-26.0]	137 (23.7) [20.2-27.2]	150 (28.3) [24.5-32.1]
Former	73 (56.2) [47.6-64.7]	269 (46.5) [42.5-50.6]	263 (49.6) [45.4-53.9]
Current	32 (24.6) [17.2-32.0]	172 (29.8) [26.0-33.5]	117 (22.1) [18.5-25.6]
Rest pain	60 (46.2) [37.6-54.7]	313 (54.2) [50.1-58.2]	272 (51.3) [47.1-55.6]
Tibial	67 (51.5) [43.0-60.1]	310 (53.6) [49.6-57.7]	303 (57.2) [53.0-61.4]
Pedal	9 (6.9) [2.6-11.3]	71 (12.3) [9.6-15.0]	70 (13.2) [10.3-16.1]
Knee popliteal			
Below	33 (25.4) [17.9-32.9]	143 (24.7) [21.2-28.3]	103 (19.4) [16.1-22.8]
Above	21 (16.2) [9.8-22.5]	54 (9.3) [7.0-11.7]	54 (10.2) [7.6-12.8]

Comparisons to the overall population revealed that the 130 patients in stage 1 had the most variation from the overall pattern of patient features. Patients in stage 1 were more likely to have isolated femoropopliteal disease (with bypass targets more often to the above- or below-knee popliteal arteries), a history of smoking and hyperlipidemia, and smaller wounds. They were less likely to have diabetes, history of stroke or myocardial infarction, dialysis dependence, and grade 2 or 3 wounds.

**Figure 2.**
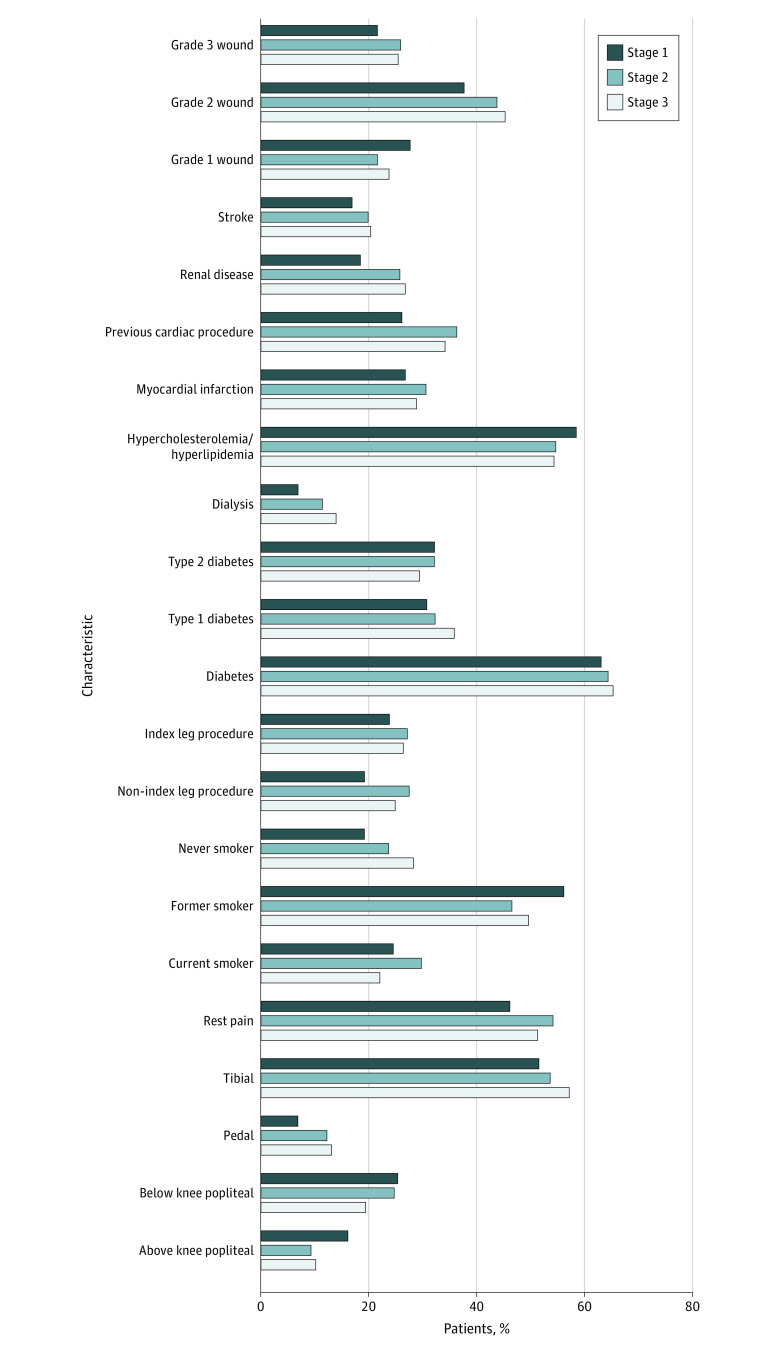
Prevalence of Characteristics in Each Stage

Patients in stage 2 (n = 578) and stage 3 (n = 530) were more similar to the cohort pattern of features but had some key differences. Patients in stage 2 were more likely to be current smokers and have a more significant medical history, including renal disease, myocardial infarction, and stroke. Of interest, patients in stage 2 were also the most likely to have grade 3 wounds. Patients in stage 3 were more likely to have never smoked, but their peripheral arterial disease seems to be more driven by type 1 diabetes, kidney disease, and dialysis dependence. This finding correlates with known patterns of atherosclerotic disease, and patients in stage 3 were more likely than the cohort mean to undergo bypasses to tibial or pedal arteries. Patients in stage 3 were also more likely to present with grade 2 and 3 wounds. The prevalence of each feature by stage is graphically presented in Figure 2.

### One-Year Mortality and Limb Loss by Stages Supervised by CLTI-Free Survival

For the entire cohort, 1-year mortality was 16.0%. When stratified by stage, mortality was as follows: 10.0% (13 of 130 observed deaths in stage 1) for stage 1, 13.5% (78 of 576 patients) for stage 2, and 20.2% (105 of 521 patients) for stage 3. We note a difference in denominators for stages 2 and 3 in the calculation of mortality that differs from the denominator used for the calculation of CLTI-free survival. Mortality information was not available for 2 patients in stage 2 and 9 patients in stage 3, but because 1-year recurrence for these patients was recorded before death, they were included in the CLTI-free survival calculations. We indicate that our calculations are based on the total number of observed deaths in the stage with the word *observed* and use this convention for calculations for the other secondary outcomes. Similarly, stratifying by stage revealed the following major limb amputation rates: 4.2% (5 of 119 observed major limb amputations in stage 1) for stage 1, 10.8% (55 of 509 patients) for stage 2, 18.4% (81 of 440 patients) for stage 3. Of those who remained alive and with an intact limb, the rates of CLTI recurrence were 9.2% for stage 1 (11 of 1 119 patients [with 11 patients excluded]), 24.9% for stage 2 (130 of 2 523 patients [with 55 patients excluded]), and 29.6% for stage 3 3 (132 of 446 patients [with 84 patients excluded]).

## Discussion

In this cohort study, topic model cluster analysis showed the ability to discern 3 distinct clusters of patients within the nested cohort of the PREVENT III data. Chronic limb-threatening ischemia–free survival at 1 year by our modeled stages was 82.3% in stage 1; 61.1% in stage 2; and 53.4% in stage 3. This method may ultimately prove superior to other methods of risk stratification in CLTI because of its ability to incorporate a wider variety of features compared with other recent risk stratification schemes, such as WIfI. Moreover, topic model cluster analysis can adjust as the prevalence and severity of risk factors change over time within a particular disease state. The malleability of topic model cluster analysis may prove more useful than prior methods of generating risk scores to evaluate modern outcome data and quality improvement projects.

In contrast to the findings of prior publications,^3,16^ wound and ischemic severity were not necessarily associated with worse outcomes, which intuitively makes sense, because not all patients with ischemic rest pain will have superior outcomes compared with those with ulcers or gangrene, and patients with less systemic disease may have better wound healing even for grade 2 and 3 wounds. This lack of strict association with ischemic and wound severity shows the importance of using CLTI-free survival as a composite end point. We should disclose that perfect discrimination between each wound grade cannot be performed retrospectively. Still, our data also show the novel promise of precisely measuring CLTI-free survival as a clinical end point. Although CLTI-free survival has been recently used by other authors,^17,18^ to our knowledge, our study represents the largest to accurately use CLTI-free survival as the primary end point. This is significant because CLTI-free survival succinctly encapsulates patient-centric outcomes, such as wound healing, and alleviation of ischemic rest pain. Further external validation of our hypothesis-generating topic cluster model will be required to confirm our findings.

Prior literature does not include the relief of ischemic rest pain as a clinical outcome measure. Moreover, prior risk stratification schemes were not able to discern subpopulations with rest pain that may have worse outcomes even though, on average, patients with rest pain have improved outcomes compared with those with ischemic ulcerations. The use of topic model cluster analysis was likely able to mathematically distinguish the subpopulations of rest pain populations that may not have superior outcomes compared with those with minor tissue loss. The difference is how topic model cluster analysis identifies the subpopulations that may have a differential risk based on the totality of their risk factors than would otherwise be suggested by an individual clinical feature, such as rest pain.

Of interest, when splitting the primary end point, we found that patients in stage 1 had a 1-year mortality of 10.0% after infrainguinal bypass, which doubled to 20.2% in stage 3. The significance of this finding is that even though the mortality rate is high overall, there is a subpopulation of patients with CLTI who are expected to achieve significantly better survival. In this subpopulation of patients in stage 1, infrainguinal bypass is safe, and endovascular therapy may not mitigate morbidity and/or mortality because of other patient comorbidities. Our study does not incorporate data regarding technical feasibility of types of revascularization, which will be required in the future. In addition, it is unclear whether these outcomes are completely generalizable, because most trial participants were White and male. Still, it is important to have criterion-standard outcomes after infrainguinal bypass to compare at least within the scientific literature. This approach permits benchmarking of current outcomes, which will be particularly important when contextualizing the results of the Best Endovascular vs Best Surgical Therapy in Patients With Critical Limb Ischemia trial.^7^

### Strengths and Limitations

There are several strengths to this study. The in-depth analysis of CLTI-free survival was long overdue and will contribute to a staging strategy to guide patients on treatment options based on outcomes more nuanced than just limb loss or death. Our novel machine learning and natural language processing methods represent a significant advance over prior literature regarding risk prediction and patient staging. This method that uses topic model cluster analysis can easily be applied to larger, more comprehensive data sets that also include specific vascular anatomy and target artery patency to further improve the predictive ability.

This research also has several limitations. A decade has passed since PREVENT III finished enrollment, and penetrance of wound care and optimal atherosclerotic medical care has changed over time, which may alter the expected outcomes of the clusters derived. Further delineation of specific distinguishing risk factors will be required also in analysis of other cohorts because the patients’ features in stages 2 and 3 were similar to those of the overall cohort mean with the exception of smoking history and dialysis dependence, which are the current differentiators between those stages. In addition, we did not have precise hemodynamic and infectious data, which limited our ability to calculate a complete WIfI score. Still, we were able to incorporate the wound severity, which is the most significant component of the WIfI score among patients who have undergone revascularization.^3^ The data were derived from a nested cohort of infrainguinal bypasses only; hence, we cannot extrapolate our results to populations undergoing endovascular and/or hybrid revascularizations.

## Conclusions

This cohort study represents one of the largest and most comprehensive mathematically derived risk stratification schemes for CLTI. By topic model cluster analysis, 3 distinct stages of 1-year CLTI-free survival were identified. This study also showed that CLTI-free survival is an end point that can be accurately measured, which shows promise in improving patient-centric outcome assessments in CLTI.
